# Feathers and Fog

**Published:** 1858-11

**Authors:** 


### FEATHERS AND FOG.

Our attention was arrested, the other day in church, by the elegance and richness of the dress of a girl of twelve years of age. There was perfect adaptation and faultless taste in every particular—nothing gaudy, nothing violent—a subdued harmony of one thing with another pervaded the whole. That child, thought we, was rearing in a family where wealth, and intelligence, and refinement, have had a home for several generations.

Within the next half hour we were in a fog. She spent part of her time in biting the edges of a costly-embossed hymn-book—then she would take it by one of the covers, and, with a jerk of the hand, throw it open and shut. She next took her laced handkerchief, made a thimble of its corner over her finger, and explored her nostril or ear, and scrutinized with considerable minuteness what she had fished out; then, with a perfect imitation of a pea-fowl, she would turn her little head one side, to look down and behind the shoulder, to see if her snow-white cape and velvet mantilla were properly adjusted; and frequently, being in a position to face nearly the entire congregation, her unveiled gaping attested her unbecoming weariness—and during all, a fretful, impatient corrugation above the eye-brow, told very plainly that she was a petted child. It was pretty certain that the dress was owing to the taste of the mother; but the manners, whence came they?

After the service was over, the child, mother, and father were united. The mother was dressed as unexceptionably as the daughter—handsome in feature, and of commanding mien, with no trace of presumption—as if she had a consciousness of a high position, was not afraid of losing it, nor felt any necessity of defining it. The father we did not observe; the upper house did, and put him down as some rich senator or governor, who had hewn out his own fortune from the stump—that it had been a marriage of calculation and adaptation, a concordat of compromises of wealth and station, with elegant birth overtaken by poverty—the joint product being a child the combination of the two, in dress elegant, in manners and face vulgar.

We really do not know what use to make of this narration, for we cannot make it "connect" with health or disease in any possible way. But, what's the use of always talking about "pills and potions." Yet it is a fact, that the young fellow who "gets that girl," will have a "bitter pill to swallow," which, likely as not, "will be the death of him."

